# Risk factors and prediction model for stone mucosa adhesion before retrograde intrarenal surgery

**DOI:** 10.20452/wiitm.2025.17973

**Published:** 2025-07-24

**Authors:** Hongliang Wei, Xiaowei Wang, Shuo Zheng, Zhong Li, Lei Wang, Wei Li

**Affiliations:** Department of Urology, First Hospital of Hebei Medical University, Shijiazhuang, China; Department of Urology Second Hospital of Hebei Medical University Shijiazhuang China

**Keywords:** adhesions, predictive model, retrograde intrarenal surgery, ureteral calculi, ureteral mucosa

## Abstract

**INTRODUCTION:**

Preoperative assessment of ureteral adhesions related to stones is crucial for choosing the right surgical approach.

**AIM:**

This study aimed to evaluate clinically significant factors for predicting the development of adhesions between the calculus and the mucosa in patients with ureteral stones undergoing retrograde intrarenal surgery.

**MATERIALS AND METHODS:**

The study included 173 patients. Ureteroscopy was performed to accurately identify the presence of adhesions between the calculus and the mucosa. Univariate and multivariable logistic regression analyses were performed to identify independent factors predicting adhesions. An alignment diagram model was developed utilizing the independent factors identified. Discrimination and calibration of the model were evaluated via the receiver operating characteristic curve and further examined via calibration curves, decision curve analysis, and cumulative intervention curves.

**RESULT:**

Adhesions between the calculus and the mucosa were confirmed on ureteroscopy in 48 patients (27.7%). Multivariable logistic regression analyses showed that ureteral wall thickness (UWT), hydronephrosis severity, perirenal fat stranding (PFS), and pain intensity were independent risk predictors of adhesions (*P* <⁠0.001; *P* <⁠0.001; *P* = 0.02; *P* = 0.02, respectively). The estimated area under the curve in the group with and without adhesions was 0.849 (95% CI, 0.742–0.832) and 0.888 (95% CI, 0.829–0.833), respectively, demonstrating an excellent predictive performance of the model.

**CONCLUSION:**

UWT, PFS, hydronephrosis severity, and pain intensity are independent risk factors for the development of adhesions between the calculus and the mucosa. Our predictive model exhibited outstanding performance, and it may help clinicians choose the most appropriate surgical method.

## INTRODUCTION

Impacted stones are difficult to manage as they cause inflammation of the adjacent ureteral mucosa, ureteral edema, and polyps, and adhere to the mucosal tissue. They are known to cause complications both during and following surgical intervention.^1^;^2^ Although not clearly defined, impacted stones prevent guidewires from passing on the first attempt, cause shock wave lithotripsy (SWL) failure, and allow the passage of opaque contrast material.^3^;^4^ Patients with impacted stones differ in terms of degree of impaction, caliber of the ureter, type of stones present, and extent of mucosal inflammation. Intravenous pyelography or computed tomography (CT) are performed to assess impacted stones, but surgeons confirm the diagnosis on ureteroscopy.^2^

During ureteroscopic lithotripsy, stones are peeled and pushed away from the stone bed to allow the guidewire to pass. The type of guidewire and method used to bypass the impacted stone are crucial for successful passage.^5^ During the surgical procedure, the inability to effectively peel and push stones away from the stone bed is indicative of adhesions between the calculus and the mucosa.

To our knowledge, however, no study has yet evaluated the risk factors related to adhesions between the calculus and the mucosa. Preoperative assessment of ureteral adhesions related to stones is crucial for determining the surgical approach. In recent years, alignment diagrams, or nomograms, have become increasingly popular and widely used in medical research evaluating the risk and prognosis of disease development.

## AIM

The objective of this study was to identify risk factors for stone mucosa adhesion prior to retrograde intrarenal surgery (RIRS), and to construct an alignment diagram, with assessment of its discriminative capability, accuracy, and clinical relevance.

## MATERIALS AND METHODS

### Study population

We retrospectively analyzed the clinical data of 201 patients with upper ureteral calculi who underwent RIRS at The First Hospital of Hebei Medical University between January 2021 and December 2024. Of those, 28 were excluded from the analysis: 13 due to incomplete data, 10 due to preoperative urinary tract infections and stent placement, and 5 due to renal fistulas. A total of 173 patients were included in the final analysis.

### Ethics

The study was approved by the Ethics Committee of The First Hospital of Hebei Medical University (V2.0/2024.02.01). Given the retrospective nature of the study, the aforementioned committee waived the requirement for informed consent.

### Inclusion and exclusion criteria

The study included 1) individuals aged 18 years or older; 2) patients with upper urinary tract calculi, confirmed based on CT images and clinical data, and not undergoing preoperative ureteral stent placement or nephrostomy; and 3) individuals who underwent RIRS.

The exclusion criteria comprised 1) individuals who presented with tumors, hematological disorders, urinary tuberculosis, or in an immunocompromised state; 2) patients diagnosed with ureteral stricture; and 3) individuals who underwent preoperative placement of a stent for renal fistula caused by urinary tract infection.

### Surgical procedures

All RIRS procedures were performed under general anesthesia and in the lithotomy position. Before RIRS, the urethra was dilated with an 8/9.5 Fr rigid ureteroscope (Karl Storz, Tuttlingen, Germany). During the surgical procedure, guidewires are usually inserted by a physician using the blind technique to bypass the stone. In cases where the guidewire cannot bypass the ureteric catheter under direct visualization, it can be retained as a safety measure.

A 9.5/11.5 Fr ureteral access sheath (UAS) was introduced over the guidewire under fluoroscopic guidance. In all cases, a domestically produced flexible ureteroscope (Guangzhou RED PINE RP-U-C12, Guangdong, China) was utilized. The stones were fragmented with holmium using a YAG laser device equipped with 200-μm laser fibers operating at energy levels of 0.8–1.2 J and frequencies of 12–15 Hz (Shanghai Reaken LaserTechnology Co., Ltd, Shanghai, China). Adherent stones were peeled off and pushed back, while nonadherent stones were fragmented and pulverized until they were amenable to spontaneous passage. After each procedure, the UAS was removed, and the presence or absence of any ureteral injuries was ascertained visually. Following the procedure, all patients underwent placement of a 6-Fr J stent which was subsequently removed 4 to 6 weeks after surgery. Stone-free status was confirmed on CT during follow-up. All procedures were performed by the same experienced endourologist.

### Data collection

Clinical data of the enrolled patients, including baseline characteristics (age, sex, body mass index [BMI], American Society of Anesthesiologists class, and pain level), preoperative comorbidities (hypertension, diabetes, hyperlipidemia, history of urinary tract stones, history of ipsilateral ureteroscopic lithotripsy, number of SWLs), and preoperative imaging and laboratory examination results (perinephric fat stranding, degree of hydronephrosis, ureteral wall thickness[UWT], CT value, blood creatinine level, stone diameter, lymphocyte count, platelet count, neutrophil count, and systemic immune-inflammation index [SII] score) were collected.

BMI was classified as follows: underweight, below 18.5 kg/m^2^; normal weight, 18.5–23.9 kg/m^2^; overweight but not obese, 24–27.9 kg/m^2^; and obese, equal to or above 28 kg/m^2^.^6^ UWT, stone diameter, perinephric fat stranding (PFS), and the presence of hydronephrosis were assessed on noncontrast CT images which were subsequently reviewed by an expert urologist. The degree of hydronephrosis was classified using a scale with scores ranging from 0 to 3, where 0 meant no hydronephrosis present in the renal collecting system, 1 corresponded to hydronephrosis confined to the renal pelvis, 2 equaled hydronephrosis characterized by significant dilation of the calyces; and 3 meant hydronephrosis with partial dilation of the calyces.^7^ The SII score was computed as the platelet count multiplied by the neutrophil count divided by the lymphocyte count. Pain severity was assessed using the visual analog scale (VAS), with scores ranging from 0 to 10 (0 = no pain, 1–3 = mild pain, 4–6 = moderate pain, 7–10 = severe pain).^8^ PFS was considered positive if there were several thick strands of soft-tissue attenuation in the perinephric space.^9^

### Statistical analysis

Continuous variables are reported as mean (SD). Categorical variables are represented as counts (percentages). The χ^2^ test or the Fisher exact test was employed for categorical variables. The *t* test was utilized for continuous variables that were normally distributed, and the Mann–Whitney test was used for skewed variables. A logistic regression model was employed for univariate and multivariable analyses. The factors that were significant (*P* <⁠0.1) in the univariate analysis were subsequently included in the multivariable logistic regression analysis. A backward stepwise method was applied for multivariable analysis, using the Akaike Information Criterion (AIC) as the selection criterion, and independent predictive factors with a *P* value below 0.05 were finally retained. A logistic regression analysis was subsequently performed exclusively on the development cohort, while bootstrap resampling (1000 iterations) was applied to assess model stability for small-sample bias.^10^;^11^ An alignment diagram model was developed utilizing the independent factors identified. The model’s discrimination and calibration abilities were evaluated via receiver operating characteristic (ROC) curves and further examined via calibration curves, decision curve analysis (DCA), and clinical impact curves (CICs). Statistical analyses were conducted using SPSS Statitics software, version 21.0 (IBM, Armonk, New York, United States) and R statistical software, version 3.5.3 (R Foundation for Statistical Computing, Vienna, Austria). A 2-tailed *P* value below 0.05 was deemed significant.

## RESULTS

The study included 173 patients (107 men and 66 women) who underwent RIRS. Mean (SD) age of the patients was 58.66 (17.9) years. Forty-eight patients (27.7%) were confirmed to have adhesions between the calculus and the mucosa during surgery, and based on that, the cohort was divided into 2 groups: the adhesion group (AG) and the nonadhesion group (NAG). Overall, 18.8% of the patients in the AG and 5.6% of the patients in the NAG had a history of ipsilateral ureteroscopic lithotripsy (intergroup comparison, *P* = 0.02; univariate analysis, *P* = 0.01).PFS was observed in 29.2% of the AG patients and 13.6% of the NAG participants (intergroup comparison, *P* = 0.03; univariate analysis, *P* = 0.02). The patients in the AG experienced less pain (intergroup comparison, *P* = 0.002; univariate analysis, *P* <⁠0.001). The proportion of severe hydronephrosis was higher in the AG than in the NAG (intergroup comparison, *P* = 0.001; univariate analysis, *P* <⁠0.001). The UWT was greater in the AG than in the NAG (intergroup comparison, *P* <⁠0.001; univariate analysis, *P* <⁠0.001), and the SII score was higher in the AG (intergroup comparison, *P* = 0.04; univariate analysis,* P* = 0.05). Other characteristics were similar in both groups.

The univariate regression analysis identified the significant (*P* <⁠0.1) factors influencing adhesion of the stone to the mucosa, and they were subsequently incorporated into the multivariable logistic regression analysis. Consequently, the aforementioned variables, along with 2 additional variables, height (intergroup comparison, *P* = 0.06; univariate analysis, *P* = 0.06) and CT value (intergroup comparison, *P* = 0.08; univariate analysis, *P* = 0.08), were included in the regression equation. The details are outlined in Table 1 and Table 2.

**Table 1:** Characteristics of patients with and without stone mucosa adhesions

Variable	Enrolled patients (n = 173)	Nonadhesion group (n = 125)	Adhesion group (n = 48)	*P* value
Age, y	58.66 (17.9)	59.96 (16.75)	55.27 (20.4)	0.12
Height, m	1.69 (0.07)	1.68 (0.07)	1.7 (0.04)	0.06
Sex				
Men	107 (61.8)	80 (64)	27 (56.2)	0.38
Women	66 (38.2)	45 (36)	21 (43.8)	
BMI				
Normal weight	108 (62.4)	82 (65.6)	26 (54.2)	0.27
Overweight	45 (26)	31 (24.8)	14 (29.2)	
Obesity	20 (11.5)	12 (9.6)	8 (16.7)	
Hypertension				
No	95 (54.9)	72 (57.6)	23 (47.9)	0.31
Yes	78 (45.1)	53 (42.4)	25 (52.1)	
Diabetes				
No	145 (83.8)	105 (84)	40 (83.3)	>0.99
Yes	28 (16.2)	20 (16)	8 (16.7)	
Hyperlipidemia				
No	98 (56.6)	73 (58.4)	25 (52.1)	0.495
Yes	75 (43.4)	52 (41.6)	23 (47.9)	
History of urolithiasis				
No	118 (68.2)	88 (70.4)	30 (62.5)	0.36
Yes	55 (31.8)	37 (29.6)	18 (37.5)	
History of ipsilateral URSL				
No	157 (90.8)	118 (94.4)	39 (81.2)	0.02
Yes	16 (9.2)	7 (5.6)	9 (18.8)	
History of ESWL				
Never	75 (43.3)	53 (42.4)	22 (45.8)	0.6
Once	76 (43.9)	54 (43.2)	22 (45.8)	
Twice	22 (12.7)	18 (14.4)	4 (8.3)	
Pain level				
No pain	53 (30.6)	29 (23.2)	24 (50)	0.002
Mild	74 (42.8)	56 (44.8)	18 (37.5)	
Moderate	45 (26)	39 (31.2)	6 (12.5)	
Severe	1 (0.6)	1 (0.8)		
ASA score				
1	104 (60.1)	77 (61.6)	27 (56.2)	0.72
2	66 (38.2)	46 (36.8)	20 (41.7)	
3	3 (1.7)	2 (1.6)	1 (2.1)	
CT-based parameters				
Stone CT value, HU	1001.04 (175.57)	986.37 (178.09)	1039.24 (164.51)	0.08
Stone diameter, mm	7.89 (1.74)	7.93 (1.77)	7.76 (1.7)	0.55
UWT, mm	2.74 (1.19)	2.37 (0.93)	3.72 (1.25)	<⁠0.001
PFS				
No	142 (82.1)	108 (86.4)	34 (70.8)	0.03
Yes	31 (17.9)	17 (13.6)	14 (29.2)	
Hydronephrosis				
Mild	77 (44.5)	62 (35.8)	15 (8.7)	0.001
Moderate	80 (46.2)	58 (33.5)	22 (12.7)	
Severe	16 (9.2)	5 (2.9)	11 (6.4)	
Laboratory parameters				
Creatinine, mg/dl	0.46 (0.1)	0.46 (0.1)	0.48 (0.09)	0.29
Neutrophil count, ×10^9^/l	4.83 (1.72)	4.79 (1.75)	4.91 (1.66)	0.7
Lymphocyte count, ×10^9^/l	1.35 (0.42)	1.37 (0.42)	1.32 (0.43)	0.48
Platelet count, ×10^9^/l	232.93 (41.12)	230.87 (41.32)	238.3 (40.55)	0.29
SII score	877.03 (356.69)	843.24 (320.77)	965.01 (428.01)	0.04

Data are presented as numbers (percentages) or mean (SD).

SI conversion factors: to convert creatinine to μmol/l, multiply by 88.4.

Abbreviations: BMI, body mass index; CT, computed tomography; ESWL, extracorporeal shock wave lithotripsy; HU, Hounsfield unit; PFS, perirenal fat stranding; SII, systemic immune-inflammation index; URSL, ureteroscopic lithotripsy; UWT, ureteral wall thickness

**Table 2:** Balance verification of the development and validation cohorts

Variable	Development cohort (n = 117)	Validation cohort (n = 56)	*P* value
Age, y	59.6 (18)	56.7 (17.8)	0.32
Height, m	1.69 (0.06)	1.7 (0.07)	0.3
Sex			
Men	72 (41.6)	35 (20.2)	0.9
Women	45 (26)	21 (12.1)	
BMI			
Normal weight	73 (42.2)	35 (20.2)	0.35
Overweight	33 (45.2)	12 (6.9)	
Obesity	11 (6.4)	9 (5.2)	
Hypertension			
No	66 (38.2)	29 (16.8)	0.57
Yes	51 (29.4)	27 (15.5)	
Diabetes			
No	100 (57.8)	45 (26)	0.39
Yes	17 (9.8)	11 (6.4)	
Hyperlipidemia			
No	67 (38.7)	31 (17.9)	0.81
Yes	50 (28.9)	25 (14.5)	
History of urolithiasis			
No	76 (43.9)	42 (24.3)	0.18
Yes	41 (23.7)	14 (8.1)	
History of ipsilateral URSL			
No	104 (60.1)	53 (30.6)	0.22
Yes	13 (7.5)	3 (1.7)	
History of ESWL			
Never	50 (28.9)	25 (14.5)	0.86
Once	51 (29.5)	25 (14.5)	
Twice	16 (9.2)	6 (3.5)	
Pain level			
No pain	32 (18.5)	21 (12.1)	0.23
Mild	52 (30.1)	22 (12.7)	
Moderate	33 (19.1)	12 (6.9)	
Severe		1 (0.6)	
ASA score			
1	73 (42.2)	31 (17.9)	0.67
2	42 (24.2)	24 (13.9)	
3	2 (1.2)	1 (0.6)	
CT-based parameters			
CT value, HU	1001 (178)	1001 (172)	0.99
Stone diameter, mm	7.99 (1.85)	7.57 (1.54)	0.12
UWT, mm	2.75 (1.16)	2.72 (1.25)	0.86
PFS			
No	97 (56.1)	45 (26)	0.68
Yes	20 (11.6)	11 (6.3)	
Hydronephrosis			
Mild	50 (28.9)	27 (16.5)	0.79
Moderate	56 (32.4)	24 (13.9)	
Severe	11 (6.4)	5 (2.9)	
Laboratory parameters			
Creatinine, mg/dl	0.47 (0.1)	0.46 (0.08)	0.39
Neutrophil count, ×10^9^/l	4.89 (1.76)	4.69 (1.62)	0.45
Lymphocyte count, ×10^9^/l	1.39 (0.42)	1.31 (0.41)	0.23
Platelet count, ×10^9^/l	232 (42.1)	234 (39.4)	0.75
SII score	871 (37)	889 (330)	0.75

Data are presented as numbers (percentages) or mean (SD).

SI conversion factors: see Table 1

Abbreviations: see Table 1

A subsequent multivariable logistic regression analysis (stepwise selection on the basis of the AIC) indicated UWT, PFS, degree of hydronephrosis, and pain level as independent risk factors for the development of stone mucosa adhesion. These findings are presented in Table 3.

**Table 3:** Results of the multiple regression analysis

Variable	Multivariable analysis	Odds ratio	95% CI	*P* value
Intercept	–	0.04	0.01–0.18	<⁠0.001
PFS	0.024	3.48	1.21–9.96	0.02
Pain severity	0.019	0.46	0.25–0.86	0.02
Hydronephrosis	<⁠0.001	3.55	1.78–7.07	<⁠0.001
UWT	<⁠0.001	4.44	2.60–7.57	<⁠0.001

Abbreviations: see Table 1

**Figure 1:**
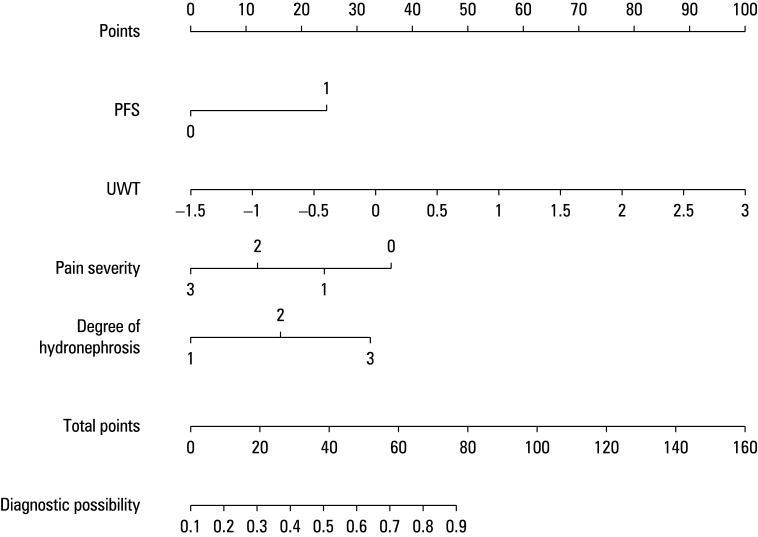
Preoperative nomogram for predicting stone mucosa adhesion

Abbreviations: see Table 1

The dataset was divided int a development cohort (70%) and a validation cohort (30%), using stratified random sampling. The balance assessment of the training and validation cohorts is presented in Table 2. A multivariable logistic regression analysis was used to construct a predictive model on the basis of the 4 aforementioned preoperative characteristics (UWT, PFS, degree of hydronephrosis, and pain level). An individualized nomogram was subsequently created, as illustrated in Figure 1.

**Figure 2:**
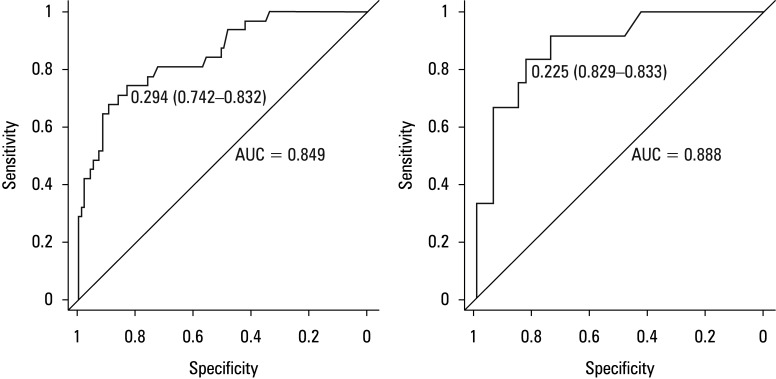
ROC curve and AUC of the model for predicting stone mucosa adhesion in the develop-ment cohort (**A**) and the validation cohort (**B**)

Abbreviations: AUC, area under the curve; ROC, receiver operating characteristic

The nomogram indicates that the overall score is derived from the individual scores of each predictive indicator. The predicted risk associated with the cumulative points is interpreted as the likelihood of stone mucosa adhesion. The model’s predictive ability was assessed through discrimination and calibration metrics. ROC curves, accompanied by area under the curve (AUC) values, were used to evaluate the model’s accuracy within both the development and validation cohorts. The estimated AUC values for the risk of impaction in these 2 groups were 0.849 (95% CI, 0.742–0.832), as illustrated in Figure 2A, and 0.888 (95% CI, 0.829–0.833), as depicted in Figure 2B, respectively, indicating the model’s excellent predictive performance.

**Figure 3:**
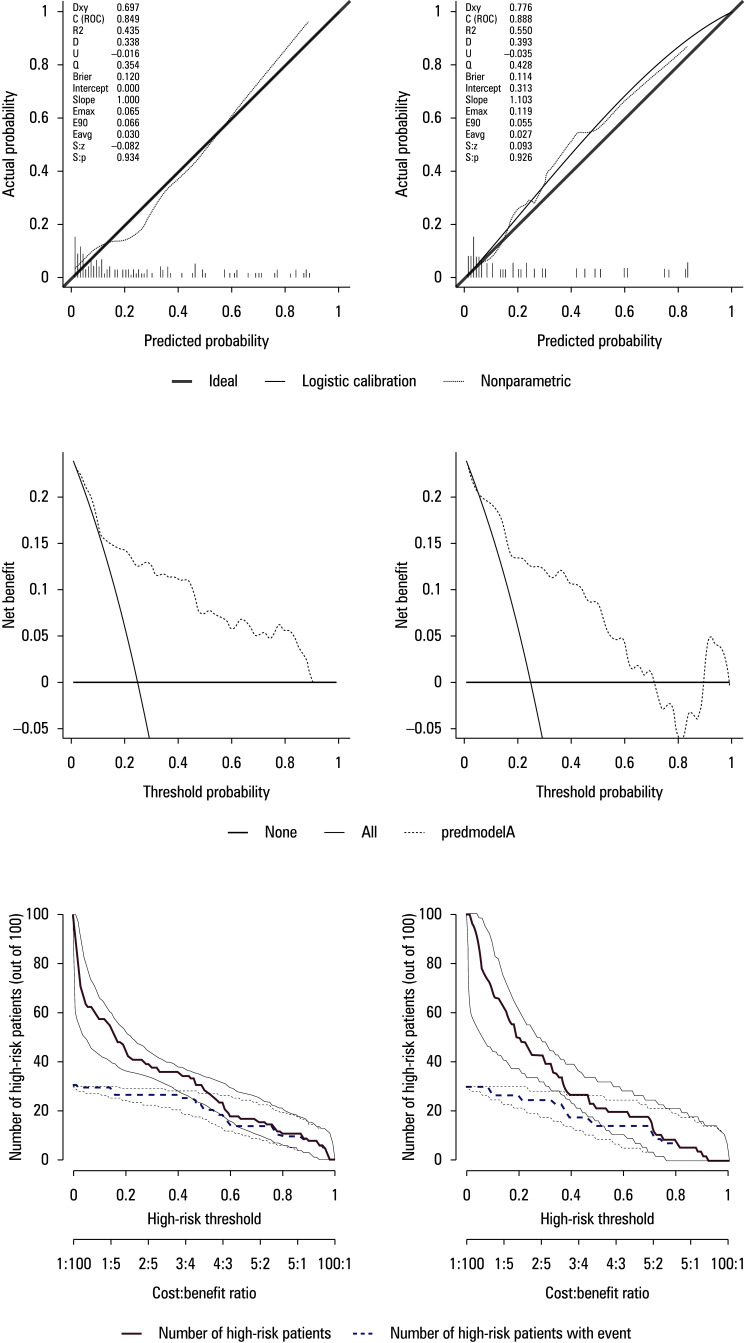
**A, B** – calibration curve of the actual vs predicted probability of the preoperative nomogram for predicting stone mucosa adhesions in the development cohort (**A**) and the validation cohort (**B**); **C, D **– decision curve analysis graphs for the predictive model in the development cohort (**C**) and the validation cohort (**D**); **E, F** – clinical impact curve of the predictive model in the development cohort (**E**) and the validation cohort (**F**)

We subsequently evaluated the performance of the model using calibration curves, DCA and CICs. The calibration curves for the current model in both cohorts exhibited robust concordance between the predicted probabilities and the actual outcomes, as depicted in Figure 3A and Figure 3B. The DCA curves, illustrated in Figure 3C and Figure 3D, were utilized to assess the clinical utility of the model. The DCA results for the development cohort demonstrated threshold probabilities ranging from 10% to 80%, whereas the validation cohort showed threshold probabilities between 10% and 70%. The CICs, presented in Figure 3E and Figure 3F, highlight the optimal clinical decision points within the high-risk threshold range of 0.2 to 0.5. These results suggest that our model has significant clinical applicability in predicting the risk of stone mucosa adhesion.

## DISCUSSION

Our findings indicate that UWT at the stone site significantly correlates with ureteral stone adhesion. The multivariable analysis demonstrated that the UWT serves as an independent pretreatment predictor of ureteral stone adhesion. In this study, UWT was assessed using noncontrast CT images, which are known for their high specificity and sensitivity in evaluating UWT.^12^

There are few studies focusing on the prediction of ureteral stone adhesion, possibly because of discrepancies in the definitions of impacted stones and the objectives of research focusing on this topic. The results of our study align with those of other studies.^13^

Ureteral hypertrophy, interstitial fibrosis, and fibrinous exudates are principal pathological changes linked to ureteral strone obstruction. These changes are believed to be caused by long-term chronic inflammation induced by stones.^14^;^15^

Chronic inflammation of the ureteral wall is frequently associated with endoscopic characteristics, such as inflammatory ureteral polyps and ureteral strictures, as noted by ^15^ Ureteral polyps increase the expression of annexin A1 and S100A9, which may play an essential role in the adhesion of calculi and polyps and the growth of calculi.^16^ The influence of these polyps on stone adhesion cannot be precisely assessed via only preoperative CT imaging. Consequently, additional research is necessary to investigate the conditions surrounding encapsulated stones.

PFS is an independent factor influencing the prediction of stone mucosa adhesion. Perinephric stranding is observed on CT images and typically results from the accumulation of fluid within the septa located in the perinephric space. In conjunction with unilateral ureteric dilation observed on spiral CT, this phenomenon has a 97% positive predictive value for the diagnosis of stone disease.^9^;^17^ The absence of PFS is associated with a 93% negative predictive value for stone disease diagnosis.^18^;^19^

Research has shown that PFS can be used as a predictive factor for spontaneous stone expulsion, and the absence of PFS is a significant predictor of spontaneous stone passage.^20^ There are few studies in which PFS is considered a predictive factor, indicating potential oversight regarding its significance in clinical practice. Our study showed that PFS may serve as a predictor for stone mucosa adhesion, highlighting the necessity for additional research to corroborate these preliminary findings.

In this study, a substantial proportion of participants (73.4%) were asymptomatic or experienced mild pain, which represents a critical area of emphasis within our research. We also found that the degree of pain was an independent prognostic factor for predicting stone mucosa adhesion. However, pain is an important symptom accompanying urinary tract stones. Interestingly, very few studies have identified pain level as a predictor for stone mucosa adhesion.

Pain intensity, as indicated by the VAS score, did not correlate with age, sex, pain characteristics, or accompanying symptoms, indicating that it may function as a reliable predictor.^21^ Patients with ureteral stones usually present with acute symptoms, chronic flank pain, hematuria, and symptoms related to urinary tract infection.^22^ The severity of renal colic is associated with the severity of obstruction rather than its degree. A higher VAS score for pain was considered indicative of the presence of a small or nonobstructing stone.^21^ In our study, 56.6% of the patients underwent SWL, indicating that patients with initial acute renal colic often autonomously choose this method of treatment first. These findings suggest that pain related to the presence of stones tends to decrease over time. The time from symptom onset to surgical intervention may serve as a more appropriate predictive variable; however, this is limited by difficulties in accurately determining the time of symptom onset in asymptomatic patients. This conclusion requires further research and verification.

Our study indicates that SWL does not contribute to adhesions formation. SWL is recognized as a primary therapeutic approach that yields positive and effective outcomes in the treatment of proximal ureteral stones, and is extensively utilized in clinical practice.^23^ Research indicates that stone-free rates associated with SWL are considerably lower and that there is potential for intraoperative and postoperative complications.^24^;^25^ This may be because SWL can exacerbate inflammation and edema of the ureteral wall, which could facilitate stone impaction.^26^;^4^ considered the presence of encapsulated stones indicative of failed SWL fragmentation. We propose that in suspected cases of impaction, SWL should be approached with caution, although our study showed that SWL is not an independent predictor of stone mucosa adhesion.

Our univariate analysis showed that a history of ureteroscopic surgery performed ipsilaterally was a significant factor associated with stone mucosa adhesion. Furthermore, it was a nonindependent predictive factor in the multivariable analysis. RIRS, whether performed with semirigid or flexible instruments, is regarded as the most prevalent technique for the treatment of upper ureteral stones. Stone-free rate for RIRS ranges from 89% to 100% when combined with laser lithotripsy.^27^;^28^ It is essential to acknowledge that complications, such as ureteral injuries, may occur due to intraoperative incidents involving the utilization of guidewires and ureteroscopes, the insertion of larger UASs, and thermal tissue damage caused by the application of a holmium laser. Additionally, stone fragments may remain after surgical procedures, and complications may occur as a result of the extraction of double J stents. Consequently, ureteral injuries may play a role in stone impaction.^5^;^29^;^30^ Therefore, in clinical practice, it is imperative to closely monitor patients who have previously undergone RIRS for any signs of ureteral injury.

Our study identified hydronephrosis as an independent risk factor for the prediction of stonemucosa adhesion. Previous studies have showed that upper urinary tract stones are associated with varying degrees of hydronephrosis, with high-grade hydronephrosis being more prevalent in patients with impacted stones.^31^;^32^;^33^ An impacted stone can cause hydronephrosis and a tortuous ureter.^34^;^35^ On the basis of our research, we speculate that hydronephrosis occurs after stone mucosa adhesion, thus the degree of hydronephrosis can predict the degree of adhesion.

In our study, the diameter of the stone was not identified as an independent predictor of stone mucosa adhesion. The correlation between stone size and the probability of spontaneous stone passage is strong. The size of the stone has a significant effect on the rate of spontaneous expulsion.Specifically, as the stone volume increases, the rate of spontaneous stone expulsion decreases. A previous study concluded that the spontaneous expulsion rate was significantly higher for stones with a diameter below or equal to 5 mm than for those measuring 5–10 mm.^20^ Larger stones of mean (SD) 11.4 (4.2) mm are impacted more often.^33^ The larger the stone is, the more likely it is to become lodged in the ureter, thereby exerting pressure on the ureteral wall. This pressure is believed to contribute to ischemia and chronic inflammation, which, in turn, may lead to ureteral edema, the formation of fibroepithelial ureteral polyps, and fibrosis, ultimately resulting in impaction.^25^;^36^;^37^;^38^ Current research, despite ongoing debate, suggests that both RIRS and percutaneous nephrolithotomy (PCNL) are effective methods in the treatment of upper ureteral stones ranging from 1.5 to 2 cm in diameter. Nevertheless, PCNL is a shorter procedure and therefore associated with a lower risk of infection and related complications.^39^ Our study did not demonstrate that stone size was a predictive factor for stone mucosa adhesion, which may be due to the relatively small mean (SD) stone diameter of 7.7 (1.7) mm in the analyzed patients.

This study has certain limitations. First, it was a small-sample, single-center retrospective study. It did not include any data on stones in pediatric patients. There is a potential of selection bias in our research, particularly at the data collection stage. Second, due to the use of different CT devices, there were variations in scan layer thickness. Finally, our prediction model has not been externally validated.

## CONCLUSIONS

We developed and validated a personalized model for predicting adhesions between the mucosa and ureteral calculi. UWT, PFS, degree of hydronephrosis, and pain level were identified as independent risk factors associated with the development of adhesions. The model exhibited outstanding predictive ability, as examined via calibration curves, DCA, and CICs, and it may guide the choice of the optimal surgical method. However, external validation is necessary to confirm its efficacy.
